# Modular Multi-Attribute Vehicle Analysis by Color, License Plate, Make and Sub-Model Using YOLO and OCR: A Benchmark Across YOLO Versions

**DOI:** 10.3390/s26092785

**Published:** 2026-04-29

**Authors:** Cristian Japhet Islas-Yañez, Viridiana Hernández-Herrera, Moisés Márquez-Olivera

**Affiliations:** Centro de Investigación e Innovación Tecnológica (CIITEC), Instituto Politécnico Nacional (IPN), Cerrada Cecati s/n Col. Sta. Catarina, Azcapotzalco, Ciudad de México 02250, Mexico

**Keywords:** multi-attribute vehicle analysis, YOLO, license-plate recognition, OCR, color classification, make and sub-model, real-time-equivalent throughput, multi-task pipeline

## Abstract

We present a modular multi-attribute vehicle analysis pipeline that integrates YOLO-based models and an OCR engine into a single workflow. The system detects vehicles, classifies color, recognizes make and sub-model, detects license plates, and extracts plate characters to generate a structured vehicle record. Vehicle detection is reported with standard metrics (precision, recall, and mAP@0.5), while license plate detection is reported at IoU = 0.3 to reflect the small-object nature of plates and downstream OCR usability. Among the evaluated versions, YOLOv8 provides the most balanced overall performance across modules, while maintaining real-time-equivalent throughput of approximately 18–22 FPS for the full pipeline on recorded traffic videos, depending on scene complexity. We emphasize module-level evaluation and runtime benchmarking; instance-level end-to-end identification across unique vehicles is defined as future work once track-based ground truth becomes available.

## 1. Introduction

The pursuit of efficient mobility has historically driven the continuous evolution of transportation systems. From their earliest forms, mobility optimization has motivated the creation of a wide variety of vehicles, ranging from individual cars to large-scale public transportation systems. These vehicles differ in shape, size, and color, which makes their automatic detection and recognition a complex task. Recent advances in computer vision and machine learning have enabled more accurate solutions to these challenges, particularly through vehicle detection and classification methods.

Accurate vehicle detection has numerous applications, including tracking, classification, and traffic monitoring [1]. Deep learning methods, especially convolutional neural networks (CNNs), have proven highly effective in image analysis, as they are inspired by the human visual system and excel at extracting visual features. Over the years, detection techniques have evolved from traditional handcrafted features to advanced frameworks capable of identifying vehicles and license plates under diverse conditions such as weather changes, illumination variations, and low image resolution. Early approaches included R-CNN, Fast R-CNN, and hybrid methods combining multiple strategies.

YOLO [2] is widely adopted for real-time object detection because it performs localization and classification in a single forward pass. In this work, we focus on YOLOv8 [3], YOLOv9 [4], and YOLOv10 [5] due to their strong trade-off between accuracy and inference speed in practical deployments, and we evaluate them under a unified protocol across all pipeline stages [6].

Despite these advances, vehicle detection remains challenging due to variability in size, orientation, illumination, and resolution in real-world environments [7]. License plate recognition adds another level of complexity, as plates vary in size, format, and resolution, making small-object detection essential [8]. Many existing studies have addressed these tasks in isolation, focusing solely on license plate recognition or on vehicle color/make detection. Reported detection rates often remain below 85% in complex scenarios [7,8], underscoring the need for an integrated, robust framework.

The outputs are consolidated into a structured multi-attribute vehicle record for each detected vehicle instance (per frame/ROI), supporting practical applications such as traffic monitoring and urban mobility analytics.

Multi-attribute records are useful in surveillance contexts where plate text can be intermittently unavailable (blur, occlusion, glare) or uncertain due to OCR errors. Color and make/sub-model provide complementary cues for: (i) filtering candidate matches when OCR yields multiple plausible strings; (ii) validating plate recognition by cross-checking with expected vehicle appearance; (iii) triaging alerts in large-scale monitoring when full identity lookup is not accessible; and (iv) supporting non-identifying analytics (traffic composition by vehicle class/color/model family) where plate text is not required.

### 1.1. Research Questions and Evaluation Objectives

This study is guided by two practical research questions:

1. RQ1. Can a unified multi-stage pipeline (vehicle → ROI → attributes → plate → OCR) reliably generate a multi-attribute vehicle record under real-world video variability (illumination changes, occlusions, and motion blur)?
2. RQ2. How do small-object-oriented detection settings affect plate localization usability for downstream OCR in recorded traffic videos?

To address these questions, we report module-level metrics for each stage (detection, classification, OCR) and runtime analysis per stage and for the full pipeline. Ablation comparisons (plate-only and partial attribute pipelines) are defined as future work once full end-to-end ground truth is available for all attributes.

### 1.2. Novelty and Contributions

(1) A modular multi-attribute vehicle analysis pipeline that produces a unified vehicle record (color + make/sub-model + plate text) from unconstrained traffic scenes.
(2) A consistent benchmarking protocol across YOLO versions (v8/v9/v10) reporting module-level metrics and runtime for each stage, including end-to-end throughput on recorded traffic videos.
(3) A privacy-aware data usage statement and reproducibility details (dataset structure, annotation rules, splits, software versions).

## 2. Related Work

Vehicle analysis in intelligent transportation systems (ITS) typically combines three components: (i) vehicle detection, (ii) attribute recognition (e.g., color and make/model), and (iii) license plate recognition (LPR). While early solutions relied on handcrafted features and classical classifiers, current state-of-the-art systems are dominated by deep learning detectors and OCR-driven pipelines.

### 2.1. YOLO-Based Detection for Vehicle and Plate Localization

Recent ITS studies widely adopt YOLO-family detectors due to their strong accuracy–speed trade-off in surveillance scenarios. For example, multi-stage systems built around YOLO-based modules have demonstrated practical vehicle and license plate recognition under low resolution and noisy acquisition conditions [9].

For LPR specifically, multiple recent implementations integrate YOLO variants with OCR engines to improve robustness across plate formats and imaging conditions; for instance, systems based on YOLOv8 combined with OCR have been evaluated for diverse plate layouts and environmental constraints [10].

### 2.2. Make/Model Recognition and Multi-Attribute Vehicle Understanding

Vehicle make and model recognition (VMMR) has grown as a complementary capability to LPR, enabling vehicle-level identification even when plates are partially occluded or degraded. A recent survey summarizes two decades of VMMR and the trend toward deep learning and fine-grained classification under challenging conditions [11].

Beyond VMMR, multi-attribute learning and recognition have been explored to jointly infer multiple cues for vehicle identity and re-identification, especially under viewpoint and lighting variability [12].

### 2.3. Integrated YOLO + OCR Pipelines

A key trend in applied ITS is the integration of detection and OCR into a single pipeline. Recent works explicitly combine YOLO detectors with OCR engines (e.g., PaddleOCR/EasyOCR) and report both recognition accuracy and runtime performance, reinforcing the relevance of reporting both recognition accuracy and runtime throughput for deployment-oriented systems [13]. Recent LPR studies also include region-specific plate systems and character-restoration approaches to mitigate OCR errors under low-quality captures [14,15].

However, most integrated pipelines remain focused on LPR alone and do not report a unified vehicle identity record that includes color and make/sub-model in addition to plate text.

Table 1 shows that most YOLO-based systems emphasize ALPR (plate detection + OCR) and commonly report recognition accuracy and runtime, but they rarely integrate plate information with additional vehicle attributes (color and make/sub-model) under a unified evaluation protocol. In parallel, multi-attribute learning is often studied within vehicle re-identification without OCR integration. This fragmentation motivates our modular multi-attribute pipeline and unified reporting of module-level metrics and inference throughput.

### 2.4. Research Gap

The literature shows strong progress in YOLO-based detection and OCR-driven LPR. Nevertheless, three gaps remain practical and under-addressed: (i) limited end-to-end evaluation of multi-attribute identification (color + make/sub-model + plate) under a single protocol; (ii) inconsistent reporting of standardized detection metrics (e.g., mAP@0.5 for vehicle detection and AP/mAP@0.3 for license plate detection), OCR accuracy, and runtime in the same study; and (iii) insufficient discussion of privacy-aware handling of license plate information and data governance.

This work addresses these gaps by proposing and benchmarking a modular pipeline that produces a unified vehicle record, reporting standardized module-level metrics and throughput, and including a privacy-aware data usage statement.

## 3. Materials and Methods

This section details the methodology of the proposed system. First, we present the overall architecture, followed by a description of each processing stage, with emphasis on convolutional neural networks (CNNs). We also provide information on the data set, software, implementation details, and evaluation metrics used to assess system performance.

### 3.1. System Architecture

**Figure 1:**
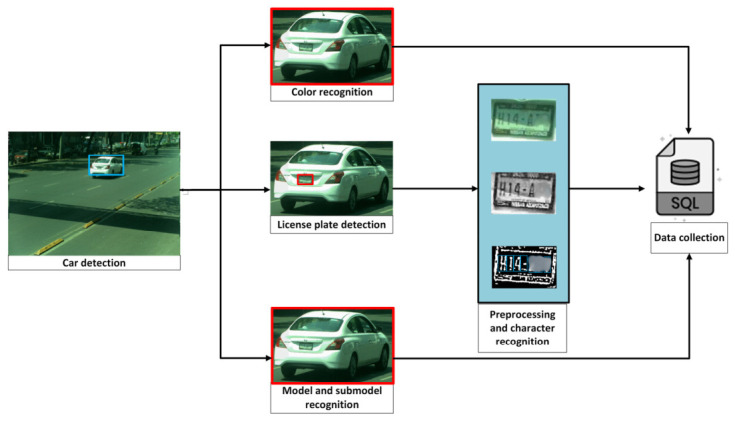
Overview of the proposed five-stage multi-attribute vehicle analysis pipeline (vehicle detection, color classification, make/sub-model recognition, plate detection, OCR, and database integration).

To recognize a vehicle by multiple attributes (color, license plate, make, and sub-model), we propose a five-stage pipeline (Figure 1): (i) vehicle detection from the full scene; (ii) color classification from cropped vehicle regions of interest (ROIs); (iii) make and sub-model recognition from the same ROIs; (iv) license plate detection followed by OCR using PaddleOCR; and (v) integration of results into a relational database.

Early ROI cropping focuses computation on relevant regions and reduces background interference for downstream stages. The modular design also enables per-stage evaluation (accuracy and runtime) and facilitates targeted optimization for deployment.

### 3.2. Vehicle Detection

In the first stage, vehicles are detected in the full scene. We use a YOLO-based detector to localize each vehicle and then crop the corresponding bounding box to generate ROIs for subsequent stages (color, make/sub-model, and license plate processing). This stage establishes the input quality for the remainder of the pipeline; therefore, we emphasize robust detection under common traffic-video variability.

**Figure 2:**
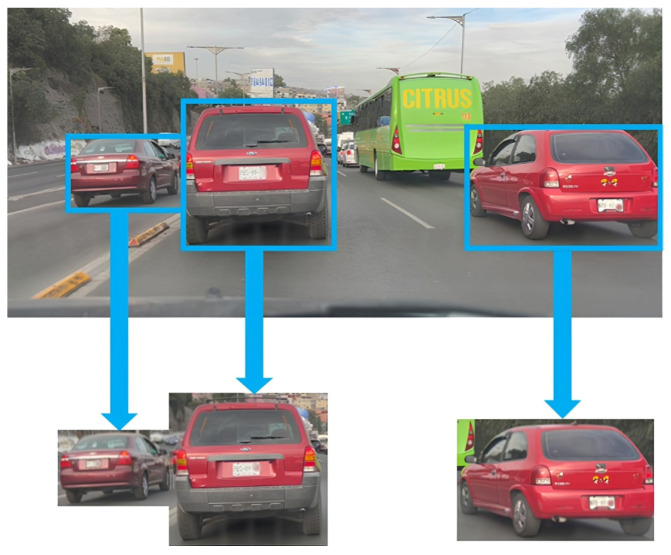
Vehicle detection outputs and ROI extraction used for downstream attribute recognition.

Step 1. Image acquisition and dataset preparation: A dataset was compiled from recorded traffic scenes captured under diverse conditions: different road environments (streets, avenues, parking lots), multiple viewpoints (front, side, rear), and varying illumination (daylight, cloudy conditions, and nighttime under artificial lighting). Each image was manually annotated with tight bounding boxes around fully visible vehicles.
Step 2. Model training: The YOLO family was selected for vehicle detection due to its efficiency in real-time scenarios. Five versions of each YOLO release are available (nano, small, medium, large, and extra-large), offering a trade-off between computational complexity and detection accuracy, as summarized in Table 2. During training, transfer learning was applied to leverage pre-trained weights, accelerating convergence and improving generalization.
Step 3. Model inference and ROI cropping: During inference, the detector produces bounding boxes for all vehicles in each frame. Each bounding box is then cropped to generate an ROI, as illustrated in Figure 2. Restricting downstream processing to ROIs reduces background clutter and preserves relevant vehicle features for subsequent attribute recognition tasks.

During real-time evaluation on an NVIDIA GeForce RTX 2070 GPU (NVIDIA Corporation, Santa Clara, CA, USA), the YOLOv8m detector achieved an average inference latency of 22 ms per frame (≈45 FPS). YOLOv9m and YOLOv10m require 29 ms and 32 ms per frame, confirming that all models sustain real-time-equivalent throughput within standard video rates. Runtime was measured on recorded traffic videos processed offline frame-by-frame; therefore, “real-time” refers to real-time-equivalent throughput rather than live camera streaming.

### 3.3. Vehicle Color Recognition

Vehicle color is a useful attribute for traffic monitoring and vehicle description in surveillance data, and it can support non-identifying analytics (e.g., traffic composition by color) when plate text is unreliable. Therefore, the key to solving this problem lies in the training set and the quality of the data used to train the CNN, which must contain a wide variety of conditions, such as variations in sunlight or artificial light, weather conditions, and others. Class balance is also crucial, as the database often contains a greater number of vehicles of certain preferred colors, such as white, silver, and black, while other colors are less common and therefore have fewer examples. This affects the neural network’s training, making it necessary to perform synthetic data augmentation or employ weighted loss functions to achieve class balance.

To address the color detection problem in this work, cropped images of the vehicles detected in stage 1 are used. Subsequently, the color of each vehicle is identified using a deep learning model. Therefore, a second network is proposed to perform this task. The model must learn visual features to distinguish between multiple color classes. Robustness at this stage is essential to reduce misclassifications caused by variations in lighting or contrast inherent in outdoor environments, which represent the greatest challenge in color detection. Both natural and artificial environmental conditions have a significant effect on the accuracy of CNNs; for example, sunlight at different times of day, weather conditions, and even street lighting are often a challenge. Therefore, one strategy is to ensure that the database includes a wide variety of these conditions.

Dataset preparation and training. We created a color-labeled dataset from the vehicle ROIs produced in Stage 1. The dataset includes variations in lighting (day/night), viewpoint, and background context to promote robustness. Class balance was improved through dataset curation and standard augmentation to reduce bias toward frequent colors.
Model inference. During inference, the trained classifier is applied to each vehicle ROI. Restricting the input to the cropped ROI reduces background interference and improves the stability of color predictions under cluttered scenes.
Result storage. Recognized colors were stored alongside other metadata for later integration with vehicle make, sub-model, and license plate data.

### 3.4. Vehicle Make and Sub-Model Recognition

The identity of a vehicle today is part of a set of characteristics; in this subsection the focus is dedicated to determining the make and sub-model, the make being the name of the manufacturer, while the sub-model is a product line (vehicle) with unique specifications of the series of cars derived from that production line, so a single make can contain a catalog of several sub-model depending on the market it seeks to impact. It is important to mention that makes can regionalize or limit the sale of their sub-models in certain countries, whether due to legislation or marketing strategies. This directly impacts the makes and sub-models being identified. This study aims to explore the detection of six sub-models belonging to three makes. These classes were selected because they are frequent in our recorded Mexico City traffic scenes and provide representative variability for fine-grained recognition. The sub-models that will be the subject of this research are Nissan_Versa, Nissan_Tiida, Chevrolet_Aveo, Volkswagen_Seat, Volkswagen_Vento and Volkswagen_Jetta.

At this stage, the challenge lies in the manual search and labeling of the selected sub-models, with the aim of creating a balanced database for each category. This will enable CNN to associate visual patterns such as headlight shapes, grilles, logos, vehicle morphology, and other characteristics. The general strategy for addressing this issue is presented below:

Dataset preparation and training. A labeled dataset of vehicle ROIs was compiled for the selected classes, aiming to maintain balanced coverage across categories. The training data include variation in viewpoint (rear/side), illumination (day/night), and vehicle pose. The classifier learns discriminative visual cues (e.g., rear-light shape, trunk geometry, emblem location, and body proportions) that support fine-grained recognition.
Model inference. The trained model was applied to the cropped ROI of each detected vehicle, focusing exclusively on relevant regions and reducing background interference.
Result storage. Predicted make and sub-model attributes were stored together with color and license plate results, forming part of a unified vehicle record.

### 3.5. License Plate Detection and Recognition

This stage provides a strong vehicle identifier by detecting and recognizing license plate characters. The process consists of two phases: (a) license plate detection and preprocessing, and (b) character recognition using OCR. The license plate is located within the original vehicle detection; subsequently, the license plate region is cropped, preprocessed to improve clarity, and processed with PaddleOCR to extract alphanumeric information using optical character recognition (OCR).

**Figure 3:**
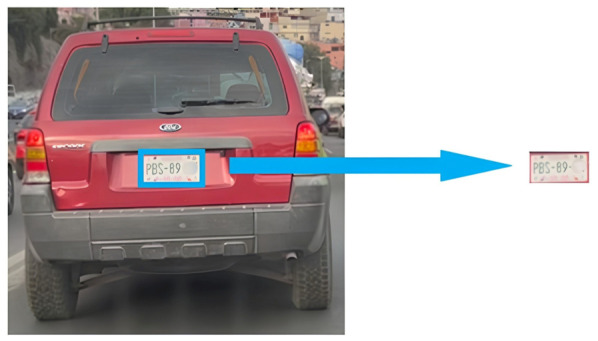
License plate localization within vehicle ROIs and plate-region cropping for OCR.

**Figure 4:**
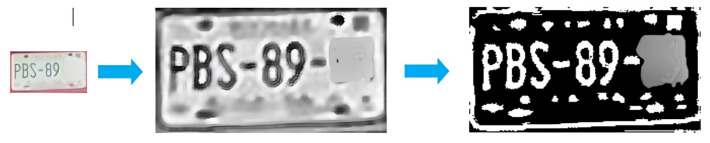
License plate preprocessing steps (denoising, grayscale conversion, adaptive thresholding, and contour filtering) to improve OCR robustness.

**Figure 5:**
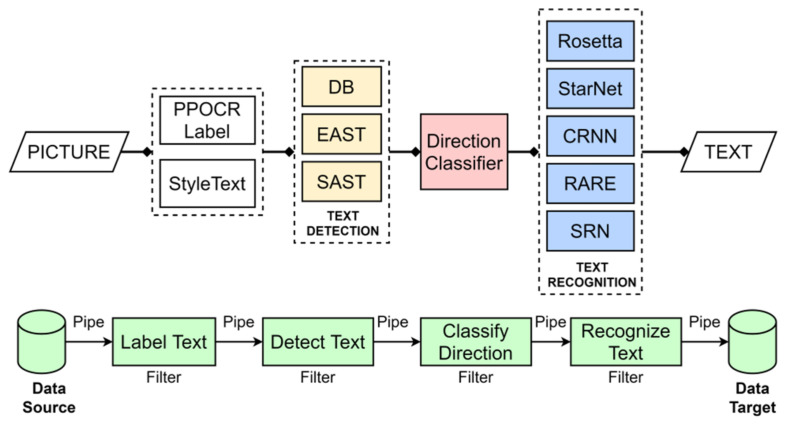
PaddleOCR pipeline used for text detection and recognition in the proposed system [16].

Step 1. Dataset and training. A license plate dataset was manually annotated under diverse conditions (illumination, viewpoint, plate type, and resolution). A YOLO-based detector was trained to localize plates by learning plate-specific cues such as rectangular borders, high-contrast character regions, and edge patterns.
Step 2. Detection and cropping. During inference, the trained model generated bounding boxes that localized plates within vehicle images. ROIs were then cropped to isolate the plate (Figure 3).
Step 3. Preprocessing pipeline. Preprocessing is applied to improve OCR robustness under blur, reflections, and low illumination. The pipeline includes: noise reduction using median and Gaussian filtering; grayscale conversion to simplify intensity structure; adaptive thresholding to enhance character–background contrast (Figure 4); and contour-based filtering to suppress non-character regions.
Step 4. PaddleOCR [16] (workflow is illustrated in Figure 5), was used in a two-stage setting (text detection + text recognition) to extract alphanumeric strings from the preprocessed plate ROI using the default detection/recognition pipeline for Latin alphanumeric characters. OCR outputs returning empty or unstable strings were discarded. We then apply rule-based post-processing to reduce common confusions (e.g., ‘0’ ↔ ‘O’, ‘1’ ↔ ‘I’/‘l’) and validate the final string against plausible alphanumeric formats. When formatting cues are available, the plate is categorized as local, foreign, or taxi.

**Figure 6:**
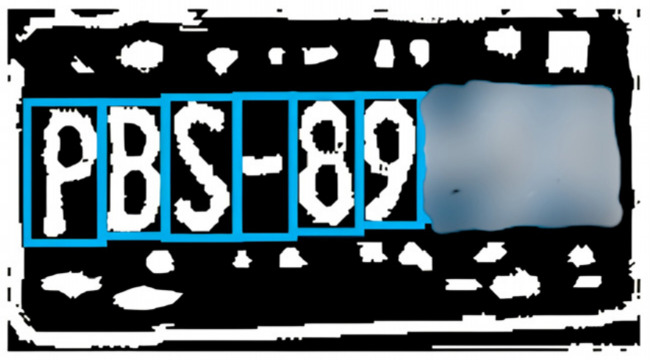
PaddleOCR text detection and recognition outputs on the preprocessed license plate ROI.

Figure 6 shows the result of using PaddleOCR on the preprocessed image, whose objective is to detect and interpret the characters.

### 3.6. Integration of Results

The final stage consolidates the outputs of all models into a structured database. For each processed frame, the system stores the detected vehicle attributes—color, make, sub-model, license plate string, and plate type—together with a timestamp. A MySQL relational database is used to support efficient storage, indexing, and retrieval for downstream ITS applications.

1. Processing and storage. Each frame generates a record containing: (i) a vehicle record ID, (ii) the recognized plate string, (iii) plate type (e.g., local/foreign/taxi), (iv) color, (v) make/sub-model, and (vi) processing date and time.
2. Database design. A central table was implemented to integrate all vehicle attributes (Table 3), enabling efficient storage and retrieval.
3. Automation and queries. Data insertion is fully automated, minimizing human errors and ensuring timely availability. The system supports fast queries (e.g., vehicles detected at each time or filtered by confidence level). Maintenance includes duplicate removal, database indexing, and periodic backups to preserve data integrity.

### 3.7. Dataset

To train the different YOLO models, we built a custom dataset from recorded urban traffic scenes captured in Mexico City using traffic monitoring/surveillance cameras (intersections and avenues). The dataset contains (i) full-scene images with multiple vehicles and (ii) cropped ROIs derived from vehicle detection, which are used for color, make/sub-model, and license plate processing. The dataset is restricted due to privacy considerations related to license plate information.

**Figure 7:**
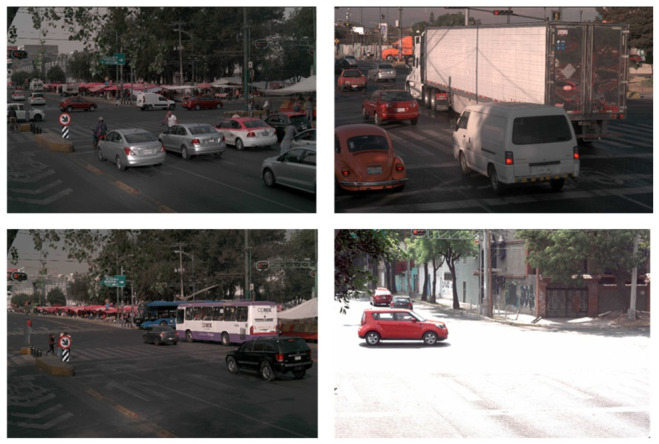
Representative full-scene images from the custom Mexico City traffic dataset used for vehicle detection.

**Figure 8:**
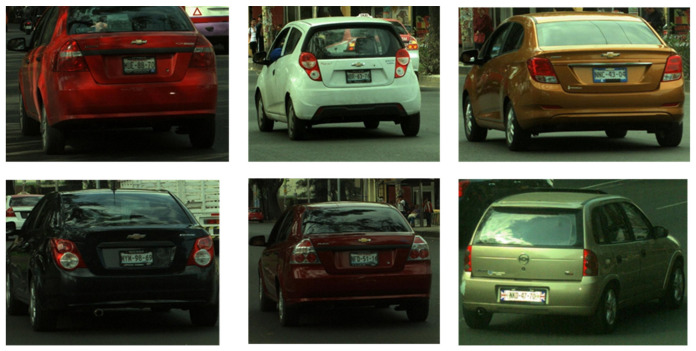
Example vehicle ROIs cropped from full scenes, used to train color, make/sub-model, and license plate modules.

The images are organized on two levels:

- Full scenes: Panoramic views containing multiple vehicles in real road context (intersections, lanes, traffic flow), with natural variability such as shadows, reflections, occlusions, and varying vehicle–camera distance (Figure 7).
- Vehicle ROIs: Cropped vehicle regions derived from full scenes, used to train color classification, make/sub-model recognition, and plate detection (Figure 8).

This dataset is suitable because it reflects real-world variability, making the model more robust against

- Changes in daylight/shadow lighting, glare, and reflections.
- Different distances and resolutions.
- High traffic density and partial visibility (unlabeled occluded cases may appear in scenes).
- Subtle differences between similar sub-models.

Data acquisition and scope. Images were obtained from recorded traffic videos collected at ten intersections/avenues in Mexico City. Frames were extracted at fixed sampling intervals and stored as RGB images. The dataset includes variability across time of day (day/night), lighting, traffic density, and viewpoint (rear/lateral), with emphasis on rear views to favor plate visibility.

Unique vehicles and identity granularity. Since the same vehicle can appear across multiple consecutive frames, the dataset contains repeated instances of the same physical vehicle. In this manuscript, annotations are performed at the image-instance level (per frame/crop). A future extension will add instance-level vehicle identity (track-based) to report end-to-end identification rates per unique vehicle.

The complete database contains 19,894 images generated from 10 different intersection and avenue locations in Mexico City. However, a meticulous selection process was carried out to avoid issues such as scene duplication resulting from the interval between shots and heavy traffic, which yields very similar images. The characteristics and number of images that were carefully selected to balance the proposed classes at each stage and with which each of the proposed CNNs trained are shown in Table 4.

Annotation protocol. All datasets were manually annotated using MakeSense.ai. Bounding boxes were drawn tightly around the target objects following consistent rules. For vehicle detection, the full visible vehicle body was enclosed. For license plate detection, the plate rectangle was annotated including the plate border when visible. Occluded objects were not annotated; vehicles or plates that were partially occluded or not clearly delineated were excluded from labeling.

Annotations were exported in YOLO format (class_id, x_center, y_center, width, height normalized by image width/height). Detection datasets used a single-class label per task (“vehicle” and “license plate”), while attribute recognition used multi-class labels (color: 11 classes; make/sub-model: 6 classes).

Training/validation splits. All task-specific datasets were split into training and validation subsets as reported in Table 4. Metrics are reported at the module level (vehicle detection, plate detection, color, make/sub-model, OCR) using the corresponding labeled subsets. Because the dataset is organized at the image-instance level (per frame/crop), repeated appearances of the same physical vehicle across consecutive frames may occur.

### 3.8. Experimental Design and Baselines

To address the research questions stated in Section 1, we evaluate the system at two levels: (i) task-level performance for each module (vehicle detection, attribute classification, plate detection, OCR), and (ii) overall pipeline throughput on recorded traffic videos; end-to-end identification across unique vehicles is reserved as future work pending track-based ground truth.

We compare the full pipeline against the following simplified baselines (ablations), evaluated under the same protocol and test split:

- B1 (Plate-only): license plate detection + OCR.
- B2 (Attributes + plate, no color): make/sub-model + plate detection + OCR.
- B3 (Attributes + plate, no make/sub-model): color + plate detection + OCR.
- Full pipeline: color + make/sub-model + plate detection + OCR.

The following ablations are defined to enable end-to-end validation once complete ground truth is available for all attributes: (B1) plate-only, (B2) make/sub-model + plate (no color), and (B3) color + plate (no make/sub-model). In the current manuscript, we report module-level performance and runtime and discuss how these components support multi-stage pipeline feasibility in real traffic videos.

### 3.9. Evaluation Metrics

For vehicle detection, we report standard detection metrics (precision, recall, and mAP@0.5). For license plates, we report detection performance at IoU = 0.3 due to the small-object nature of plates and the downstream OCR requirement, where slight localization offsets can still yield correct text recognition. For attribute classification (color and make/sub-model), we report accuracy and macro-F1. For OCR, we report character-level accuracy and exact-match plate accuracy.

End-to-End Vehicle Identification Rate (E2E-VIR). We define a vehicle as correctly identified only if the predicted tuple (color, make/sub-model, license plate string) matches the ground truth for the same vehicle instance. This metric is defined for future end-to-end evaluation once full instance-level annotations are completed.

Software and availability. Ultralytics YOLO was used via the official Ultralytics repository (https://github.com/ultralytics/ultralytics; accessed on 10 October 2024). OCR was performed with PaddleOCR using the official repository (https://github.com/PaddlePaddle/PaddleOCR; accessed on 25 October 2024). Manual annotation was carried out with MakeSense.ai (https://www.makesense.ai/; accessed on 30 October 2024). The relational database was implemented using MySQL (https://www.mysql.com/; accessed on 20 November 2024).

## 4. Results

This section presents qualitative examples and quantitative metrics for each stage of the pipeline—vehicle detection, color, make/sub-model, and license plate OCR—highlighting performance across diverse scenes and reporting precision, recall, and confusion matrices to enable a consistent module-level evaluation across stages and an overall throughput benchmark for the complete pipeline.

Inference speed was measured for each YOLO configuration to validate real-time suitability. Across all models, the full multi-stage pipeline—comprising vehicle detection, color recognition, make/sub-model classification, and license-plate OCR—achieved total processing times between 42 and 58 ms per frame depending on the scene complexity and model size. The YOLOv8-based configuration maintained an average of 18–22 FPS, while YOLOv9 and YOLOv10 variants reached 14–17 FPS. Videos are used as a source of diverse frames and for throughput measurement; temporal fusion/tracking is not exploited in the current version and is considered future work.

For vehicle detection, we report precision, recall, and mAP@0.5. For license plates, we report AP@0.3/mAP@0.3 to reflect small-object detection and OCR usability, computed with the standard 0–1 recall integration (101-point interpolation). For classification tasks (color; make and sub-model) we report top-1 accuracy, macro-precision, macro-recall, and macro-F1, together with confusion matrices. Unless otherwise stated, inference thresholds were confidence = 0.25 and NMS IoU = 0.45. For evaluation, we use mAP@0.5 for vehicle detection and AP/mAP@0.3 for license plate detection to reflect small-object/OCR usability.

Validation Focus. Because complete end-to-end ground truth (color + make/sub-model + plate string per unique vehicle instance) is not available for all samples, the current study focuses on: (i) module-level performance for vehicle detection, plate detection, color recognition, make/sub-model recognition, and OCR; (ii) runtime analysis per stage and total pipeline throughput on recorded traffic videos; and (iii) qualitative evidence of generating a multi-attribute vehicle record in real-world scenes.

The ablation comparisons defined in Section 3.8 are reserved as future work once full instance-level annotations are completed.

### 4.1. Vehicle Detection Model

The first detector was trained on a dataset of 2383 vehicle images collected under diverse traffic scenes and illumination conditions. The dataset was split into 63% for training and 37% for validation to enable early performance monitoring and hyperparameter tuning. A second training phase applied transfer learning on an additional dataset of 2423 images, reusing pretrained weights to accelerate convergence and enhance generalization. In this phase, the data were split into 80% for training and 20% for validation, prioritizing exposure to new examples while preserving sufficient validation samples for overfitting control.

Across both phases—comprising approximately 11,000 training and validation iterations—the adopted strategy reduced overall training time, mitigated overfitting, and stabilized performance prior to integrating the detector into the full multi-stage pipeline. This step provided a robust foundation for subsequent recognition tasks, ensuring that vehicle detection served as a reliable baseline for the multi-attribute recognition system.

#### 4.1.1. Vehicle Detection Training Metrics

For the task of vehicle detection, the hyperparameters that were proposed for the training of the CNN of the YOLO model after experimental tests and considering the best performance obtained are those shown in Table 5.

Background handling and unlabeled vehicles. In unconstrained traffic scenes, not all vehicles present in a frame were labeled. This can inflate apparent background → vehicle confusion because unlabeled vehicles may be counted as background during evaluation. Therefore, vehicle detection metrics are reported on the curated labeled subsets described in Table 4, and we treat the unlabeled-vehicle effect as a limitation when interpreting background errors.

The performance of the vehicle detection models was evaluated using confusion matrices and training curves (classification loss, precision, and recall), enabling a comprehensive assessment of accuracy and learning dynamics.

**Figure 9:**
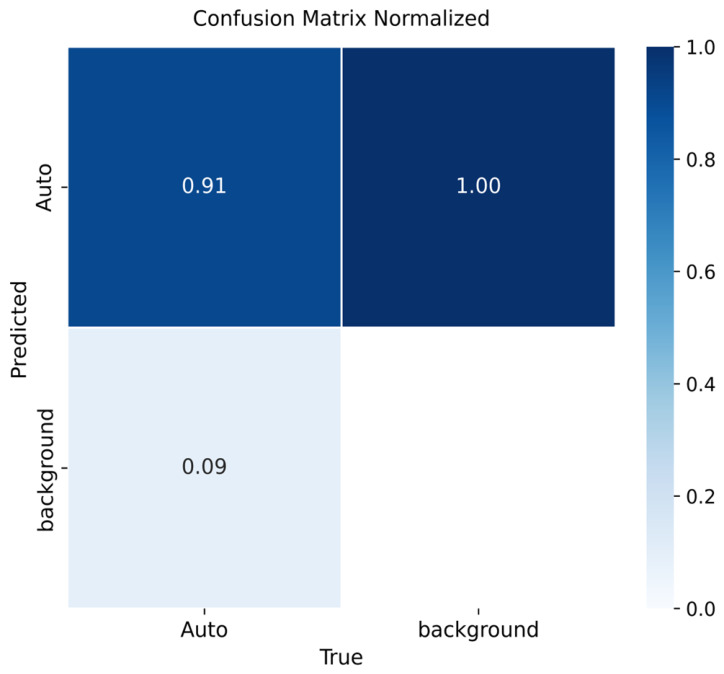
Confusion matrix for vehicle detection using YOLOv8m on the validation set.

**Figure 10:**
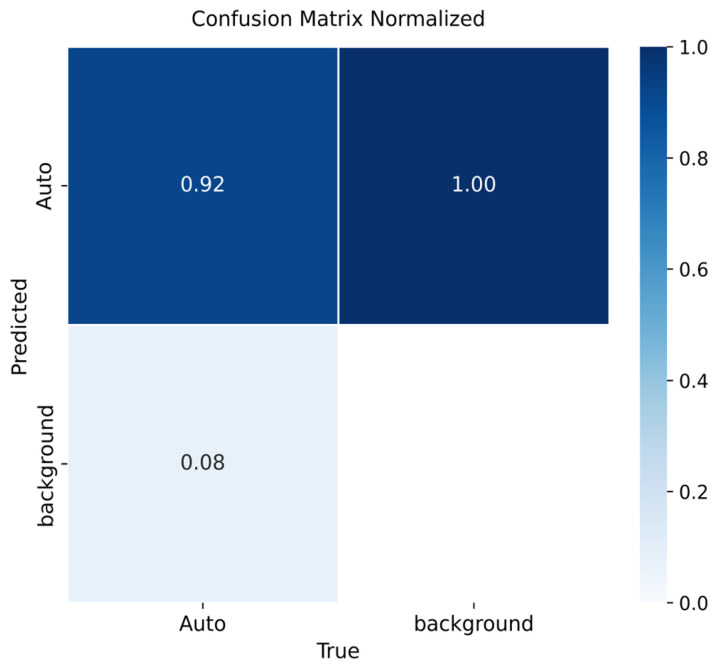
Confusion matrix for vehicle detection using YOLOv9m on the validation set.

**Figure 11:**
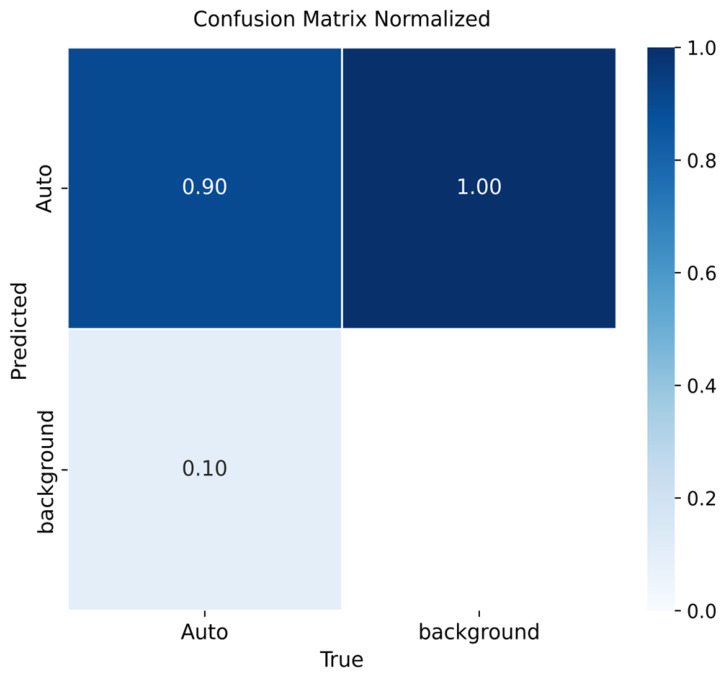
Confusion matrix for vehicle detection using YOLOv10m on the validation set.

Figure 9, Figure 10 and Figure 11 summarize the detection performance of YOLOv8m, YOLOv9m, and YOLOv10m, respectively.

For YOLOv8m (Figure 9), the model achieved 91% precision for vehicle detection. Background regions in the validation set were systematically predicted as vehicles (100%), which is plausibly explained by unlabeled vehicles present in background areas. Additionally, 9% of vehicles were misclassified as background, yielding occasional false negatives.

For YOLOv9m (Figure 10), precision reached 92%, the highest among the three versions. Background regions were again entirely misclassified as vehicles, and 8% of vehicles were missing, indicating a slightly lower false negative rate than YOLOv8m.

For YOLOv10m (Figure 11), precision was 90%. All background areas were erroneously labeled as vehicles, while 10% of vehicles were misclassified as background, suggesting higher sensitivity to adverse illumination and lower resolution.

**Figure 12:**
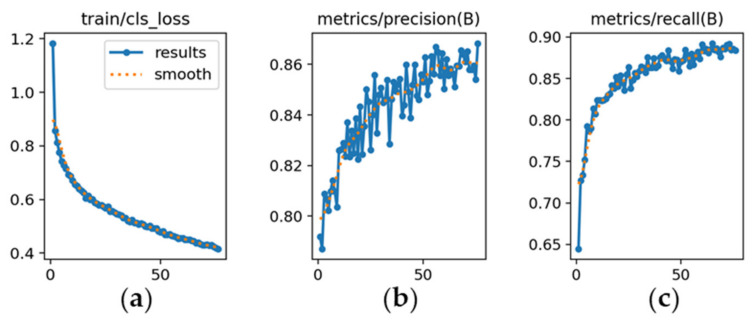
Training curves for vehicle detection with YOLOv8m (*x*-axis: epochs): (**a**) classification loss; (**b**) precision; (**c**) recall.

**Figure 13:**
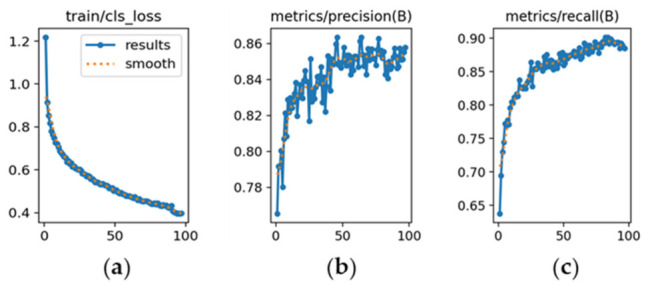
Training curves for vehicle detection with YOLOv9m (*x*-axis: epochs): (**a**) classification loss; (**b**) precision; (**c**) recall.

**Figure 14:**
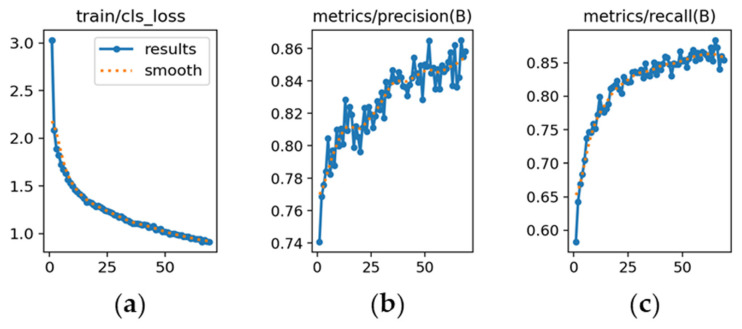
Training curves for vehicle detection with YOLOv10m (*x*-axis: epochs): (**a**) classification loss; (**b**) precision; (**c**) recall.

The learning dynamics are detailed in Figure 12, Figure 13 and Figure 14, and each subfigure is explicitly referenced below to ensure clarity.

YOLOv8m (Figure 12): Figure 12a shows a steady decrease in classification loss, confirming stable convergence without abrupt fluctuations. Figure 12b depicts precision rising from 0.78 and stabilizing around 0.86. Figure 12c illustrates recall increasing from 0.60 to approximately 0.90, indicating high sensitivity.

YOLOv9m (Figure 13): Figure 13a demonstrates a consistent reduction in loss throughout training. Figure 13b shows precision improving from 0.76 to about 0.85 by the final epoch. Figure 13c presents recall growth from 0.63 to 0.89, reflecting slightly higher sensitivity than YOLOv8m.

YOLOv10m (Figure 14): Figure 14a indicates higher initial loss and slower convergence relative to the other versions. Figure 14b shows precision starting at 0.74 and stabilizing near 0.85 after approximately 70 epochs. Figure 14c highlights recall increasing from 0.57 to 0.85, confirming acceptable sensitivity despite slower adaptation.

Across versions, vehicle detection performance was high (mAP@0.5): YOLOv8m = 0.91, YOLOv9m = 0.92, YOLOv10m = 0.90. YOLOv9m achieved the best precision–recall trade-off, whereas YOLOv8m showed smoother convergence and robust generalization. A consistent limitation was background being flagged as “vehicle”; adding background-only images is expected to reduce these false positives.

#### 4.1.2. Vehicle Detection Predictions

**Figure 15:**
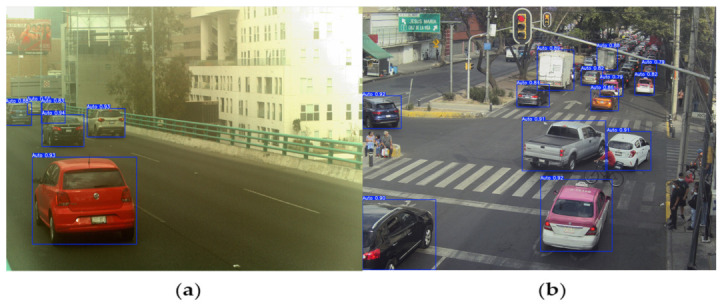
Qualitative vehicle detection examples using YOLOv8m: (**a**) rear and lateral views; (**b**) wide-angle scenes and partially visible vehicles.

After completing the training process, the YOLOv8m detector was evaluated on unseen data. Representative examples are shown in Figure 15. The model consistently identified visible vehicles with confidence scores above 80%, confirming generalization beyond the training set.

In Figure 15a, rear and lateral viewpoints are correctly detected, including cases with partial occlusions and dense traffic, indicating that the model captured structural features (silhouettes and contours) that remain stable across common perspectives. In Figure 15b, more challenging scenes—wide-angle perspectives, moving vehicles, and partially visible targets—were also handled reliably; the predicted bounding boxes closely matched the ground truth, demonstrating robustness under real-world conditions.

The predictions confirm reliable detection performance across diverse viewpoints and environments. This strong baseline enables downstream stages—color, make, sub-model, and license plate recognition—to operate on high-quality regions of interest, reinforcing the full multi-stage pipeline.

### 4.2. Vehicle Color Recognition Model

The second stage of the pipeline involved the classification of vehicle colors. The model was trained on a dataset of 2245 images, carefully labeled by color and collected under diverse conditions to ensure robustness. Images were captured under sunny, cloudy, and nighttime lighting, from multiple viewpoints (frontal, lateral, and rear), and across different environments such as urban streets, parking lots, and avenues.

The dataset covered the following color classes: yellow, blue, white, brown, maroon, orange, black, silver, red, green, and taxi. To enable monitoring of model generalization, the dataset was split into 78% training and 22% validation. Training was performed over 150–170 epochs with iterative validation, allowing gradual improvements in classification accuracy and robustness against variations in illumination and contrast.

#### 4.2.1. Color Recognition Training Metrics

For the task of determining the color of the previously detected vehicle, the hyperparameters proposed for training the CNN of the YOLO model after experimental testing and considering the best performances obtained are shown in Table 6.

For a fair comparison, the same hyperparameter configuration was used for YOLOv8m, YOLOv9m, and YOLOv10m in this task (Table 6).

**Figure 16:**
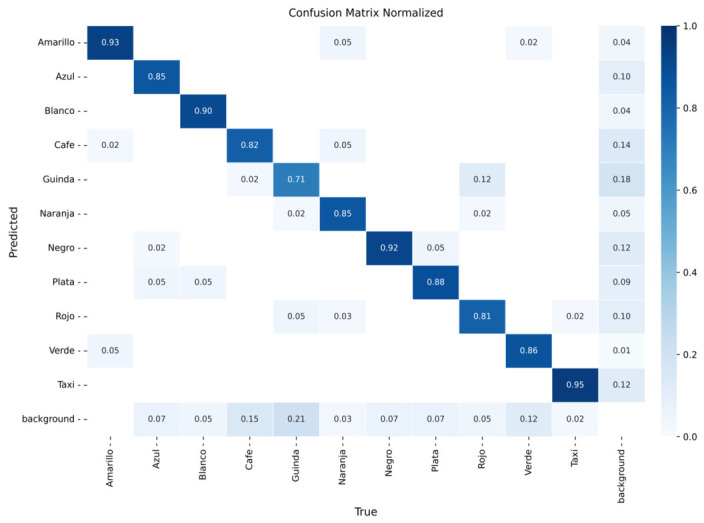
Confusion matrix for vehicle color recognition using YOLOv8m on the validation set.

**Figure 17:**
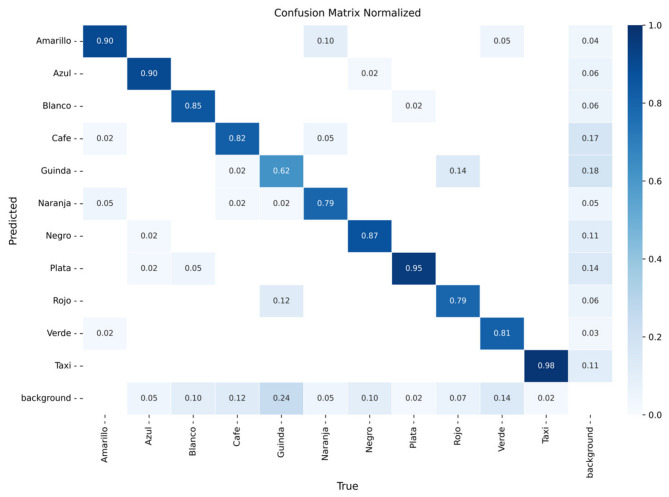
Confusion matrix for vehicle color recognition using YOLOv9m on the validation set.

**Figure 18:**
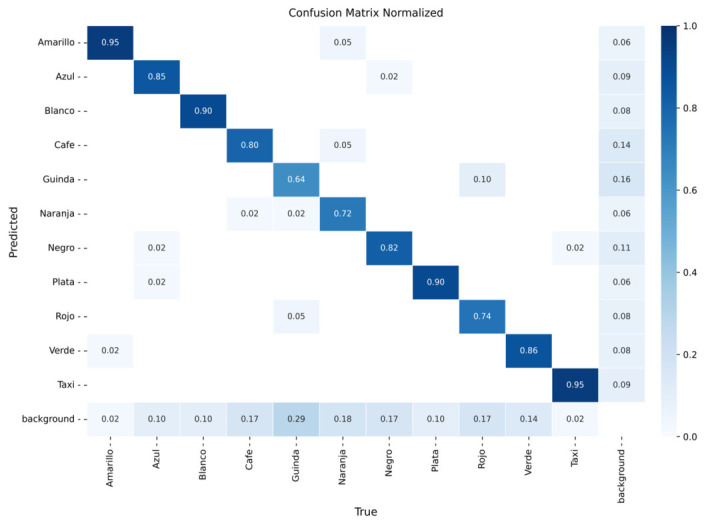
Confusion matrix for vehicle color recognition using YOLOv10m on the validation set.

**Figure 19:**
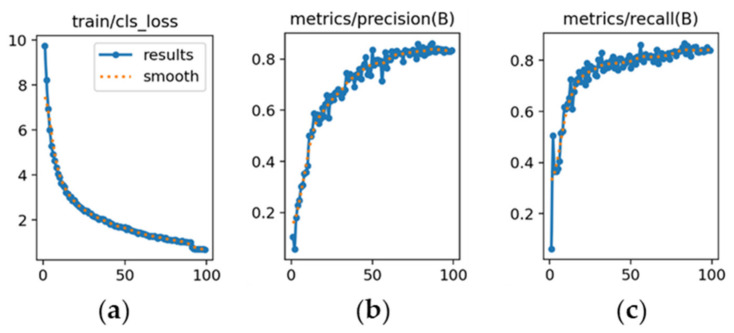
Training curves for vehicle color recognition with YOLOv8m (*x*-axis: epochs): (**a**) classification loss; (**b**) precision; (**c**) recall.

**Figure 20:**
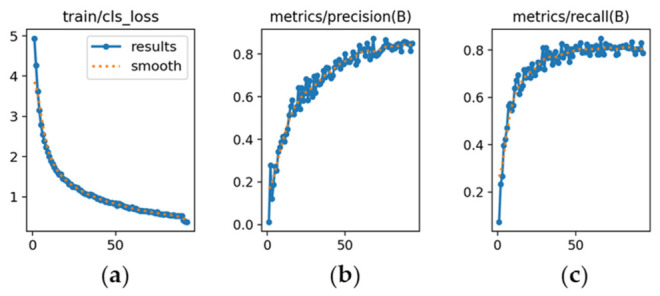
Training curves for vehicle color recognition with YOLOv9m (*x*-axis: epochs): (**a**) classification loss; (**b**) precision; (**c**) recall.

**Figure 21:**
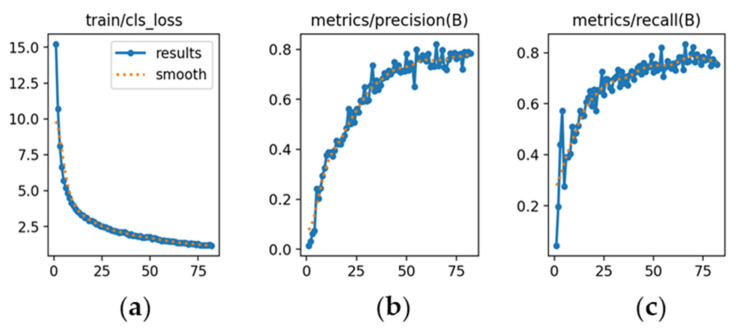
Training curves for vehicle color recognition with YOLOv10m (*x*-axis: epochs): (**a**) classification loss; (**b**) precision; (**c**) recall.

The models were evaluated using confusion matrices and training curves for classification loss, precision, and recall. Figure 16, Figure 17 and Figure 18 present the confusion matrices for YOLOv8m, YOLOv9m, and YOLOv10m, respectively, while Figure 19, Figure 20 and Figure 21 illustrate their training dynamics.

YOLOv8m (Figure 16): Achieved high accuracy for yellow (93%), black (92%), and taxi (95%). Intermediate results were obtained for blue (85%), white (90%), orange (85%), and silver (88%), where misclassifications were often related to lighting variations and reflections. Lower performance was observed for maroon (71%) and red (81%), which were confused with other dark colors due to tonal similarities.

YOLOv9m (Figure 17): Reached excellent performance for silver (95%) and taxi (98%). Intermediate precision was observed for black (87%), yellow (90%), blue (90%), white (85%), green (81%), and brown (82%). The most frequent confusions occurred for maroon (62%) and orange (79%), again due to tonal variability.

YOLOv10m (Figure 18): Delivered strong accuracy for yellow (95%) and taxi (95%). Intermediate values were obtained for white (90%), silver (90%), green (86%), and blue (85%). Lower accuracy was observed for maroon (64%), orange (72%), and red (74%), with frequent confusion among similar dark shades.

The learning dynamics of each model are presented in Figure 19, Figure 20 and Figure 21.

YOLOv8m (Figure 19): Figure 19a shows a consistent decrease in classification loss, indicating stable optimization. Figure 19b illustrates precision starting at 0.78 and stabilizing around 0.86. Figure 19c shows recall rising steadily and stabilizing near 0.89, reflecting balanced precision–recall performance.

YOLOv9m (Figure 20): Figure 20a demonstrates a steady reduction in classification loss, starting from a value of 5. Figure 20b shows precision increasing and stabilizing at approximately 0.82, slightly below YOLOv8m. Figure 20c indicates recall stabilizing around 0.80, reflecting adequate sensitivity with moderate variability.

YOLOv10m (Figure 21): Figure 21a shows higher initial classification loss (~15), which decreased gradually, indicating slower adaptation. Figure 21b presents precision converging around 0.78, with a tendency to generate more false positives than the other models. Figure 21c shows recall stabilizing near 0.78, indicating lower sensitivity under challenging illumination or tonal similarity.

YOLOv8m achieved the best balance between precision and recall, while YOLOv9m demonstrated slightly lower precision but consistent performance. YOLOv10m exhibited slower convergence and lower sensitivity, particularly for colors with high tonal overlap such as maroon, red, and brown. These results highlight the importance of dataset diversity to address variations in illumination and reflections that can significantly affect chromatic classification.

#### 4.2.2. Color Recognition Predictions

**Figure 22:**
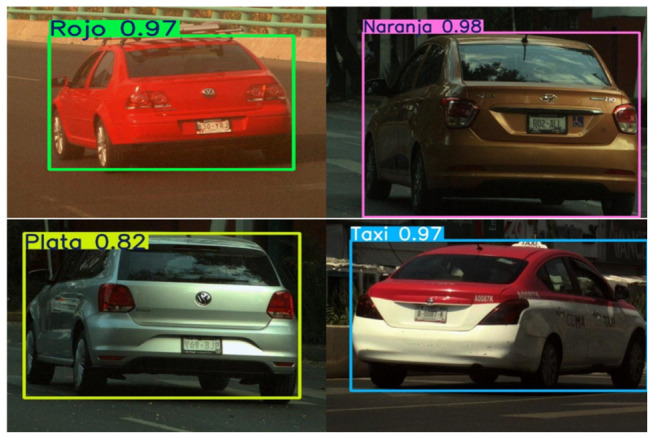
Qualitative examples of vehicle color recognition using YOLOv8m under varying illumination and viewpoints.

Representative predictions are shown in Figure 22, where the YOLOv8m model demonstrated consistent performance in identifying vehicle colors across diverse environments. Vehicles were correctly classified in different lighting conditions, including daylight, cloudy weather, and nighttime illumination, as well as from multiple viewpoints. These results confirm that the model generalizes effectively to real-world scenarios where illumination and background contrast vary significantly.

### 4.3. Make and Sub-Model Recognition Model

The third stage of the pipeline focused on the recognition of vehicle make and sub-model. For this task, YOLOv8m, YOLOv9m, and YOLOv10m were trained and evaluated for make and sub-model recognition under the same hyperparameter configuration to ensure a fair comparison.

The dataset comprised 8593 labeled images distributed into common vehicle categories (e.g., Nissan, Chevrolet, Volkswagen). The split was 86% training and 14% validation, ensuring both sufficient exposure to category variability and an adequate set for generalization testing. Images included diverse conditions such as natural and artificial lighting, multiple capture angles, and vehicles in motion as well as parked.

The model was trained for approximately 100 epochs, progressively optimizing weights to handle challenging cases such as illumination variability and partially occluding make logos. Balanced representation of all categories was maintained to minimize class bias.

#### 4.3.1. Make/Sub-Model Recognition Training Metrics

The objective of this stage focuses its efforts on the detection of the make and sub-model of the previously detected vehicles. Table 7 shows the proposed hyperparameters for training the CNN of the YOLO model versions once the experimental tests are carried out and considering the best performances obtained.

The same training hyperparameters were applied across YOLOv8m, YOLOv9m, and YOLOv10m for make/sub-model recognition (Table 7).

**Figure 23:**
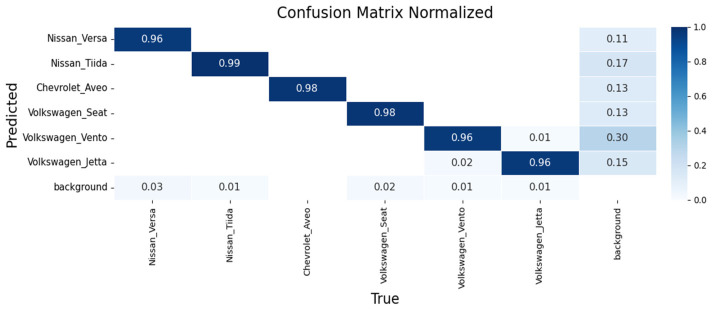
Confusion matrix for make and sub-model recognition using YOLOv8m on the validation set.

**Figure 24:**
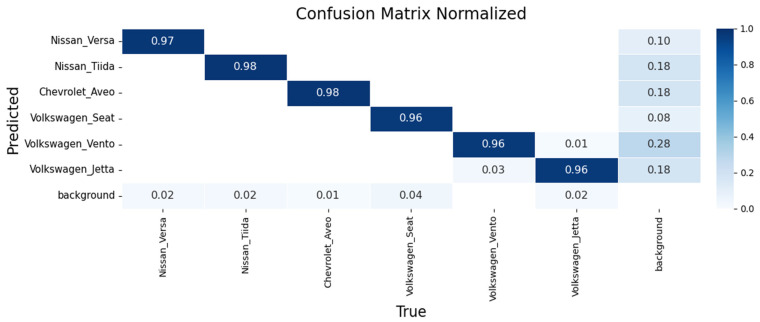
Confusion matrix for make and sub-model recognition using YOLOv9m on the validation set.

**Figure 25:**
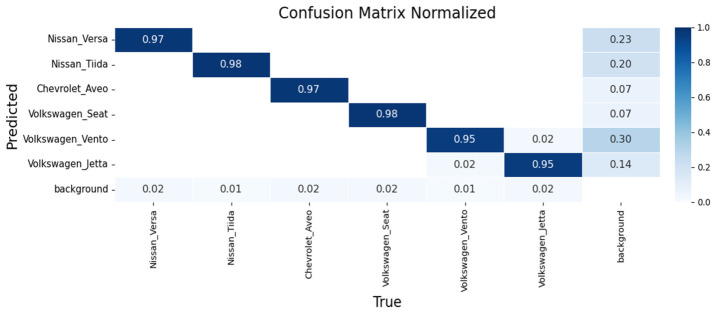
Confusion matrix for make and sub-model recognition using YOLOv10m on the validation set.

Performance was assessed using confusion matrices and training curves. Figure 23, Figure 24 and Figure 25 present confusion matrices for YOLOv8m, YOLOv9m, and YOLOv10m.

YOLOv8m (Figure 23): Achieved 96% accuracy for Nissan Versa and 99% for Nissan Tiida, demonstrating robust intra-make differentiation. Recognition of the Chevrolet Aveo was also consistent, while models from the Volkswagen group (Seat, Vento, Jetta) were classified with 94–96% accuracy, with minor confusion between Vento and Jetta due to strong design similarities.

YOLOv9m (Figure 24): Delivered slightly higher precision, with 97% for Nissan Versa, 98% for Nissan Tiida, and 98% for Chevrolet Aveo. Volkswagen models were recognized with 96% accuracy each, though confusions again occurred between Vento and Jetta.

YOLOv10m (Figure 25): Reported 97% for Nissan Versa, 98% for Nissan Tiida, and 97% for Chevrolet Aveo. Volkswagen vehicles reached 95–98%, confirming robustness but with occasional errors between Vento and Jetta under specific lighting or angles.

**Figure 26:**
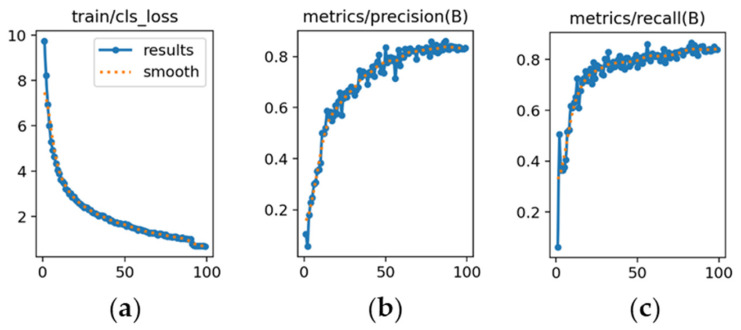
Training curves for make and sub-model recognition with YOLOv8m (*x*-axis: epochs): (**a**) classification loss; (**b**) precision; (**c**) recall.

**Figure 27:**
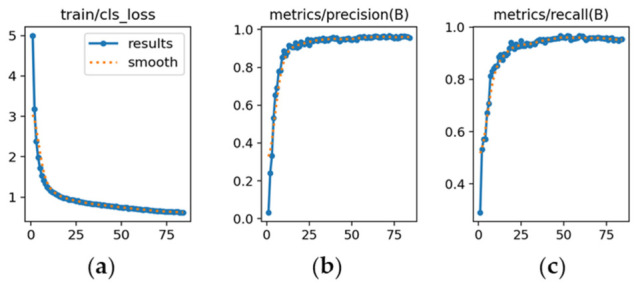
Training curves for make and sub-model recognition with YOLOv9m (*x*-axis: epochs): (**a**) classification loss; (**b**) precision; (**c**) recall.

**Figure 28:**
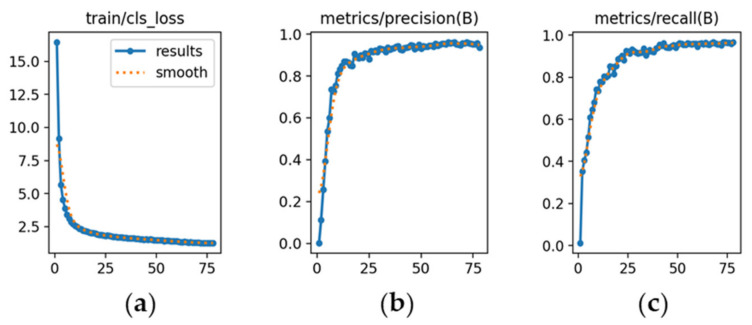
Training curves for make and sub-model recognition with YOLOv10m (*x*-axis: epochs): (**a**) classification loss; (**b**) precision; (**c**) recall.

The learning dynamics for each model are shown in Figure 26, Figure 27 and Figure 28.

YOLOv8m (Figure 26): Figure 26a illustrates a steady decrease in classification loss, reflecting consistent optimization. Figure 26b shows precision increasing above 0.90 by epoch 20 and remaining stable until early stopping (epoch 70). Figure 26c demonstrates recall surpassing 0.90 by epoch 25, indicating balanced performance.

YOLOv9m (Figure 27): Figure 27a shows loss reduction beginning at 5 and decreasing smoothly until epoch 80. Figure 27b presents precision converging near 1.0 by epoch 25 and remaining constant, reflecting excellent discriminative capability. Figure 27c shows recall following a similar trend, stabilizing near 1.0.

YOLOv10m (Figure 28): Figure 28a displays higher initial loss (17.5) that decreased gradually over 78 epochs. Figure 28b indicates precision climbing progressively to 1.0 and remaining stable. Figure 28c shows recall following the same trajectory, confirming strong sensitivity across categories.

All three models achieved high accuracy for make and sub-model recognition, with YOLOv9m and YOLOv10m slightly outperforming YOLOv8m in recall and precision. Nonetheless, YOLOv8m demonstrated smoother convergence and benefited from early stopping, avoiding overfitting. Persistent confusions between visually similar models (e.g., Volkswagen Vento and Jetta) highlight the challenge of fine-grained recognition and the importance of dataset diversity.

#### 4.3.2. Make/Sub-Model Recognition Predictions

**Figure 29:**
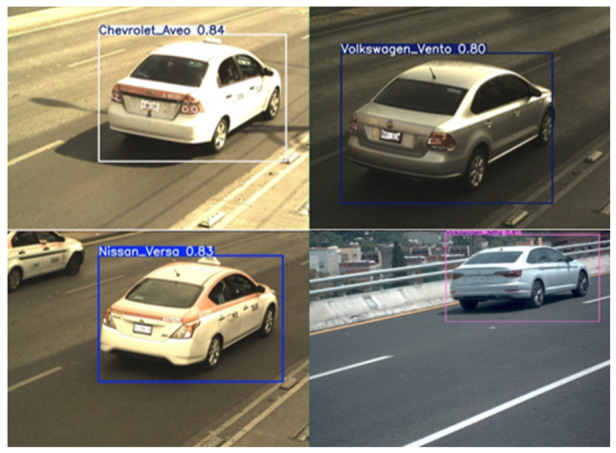
Qualitative examples of make and sub-model recognition using YOLOv8m on representative vehicle ROIs.

Representative outputs are illustrated in Figure 29. YOLOv8m was able to accurately classify vehicles from both common and visually similar categories, correctly distinguishing between Nissan Versa and Tiida, Chevrolet Aveo, and Volkswagen models. Predictions were consistent even under partial occlusions, variable illumination, and non-standard viewpoints, confirming robust generalization.

### 4.4. License Plate Detection Model

The fourth stage of the pipeline focused on license plate detection. To improve robustness, two datasets were combined: the first contained 7324 license plate images collected from different road environments worldwide, and the second contained 1200 additional samples used for transfer learning. Both datasets were split into 80% training and 20% validation.

The YOLOv8x architecture was selected due to its larger number of parameters, enabling enhanced detection of small objects such as license plates. This model was compared with YOLOv9e and YOLOv10x to evaluate performance across versions. Training was performed over several cycles, totaling ~9300 images, ensuring broad coverage of different conditions:

- Lighting variability: intense daylight, nighttime low illumination, and artificial lighting.
- Angle diversity: front, lateral, and oblique captures.
- Plate diversity: various formats, sizes, colors, and fonts.

#### 4.4.1. License Plate Detection Training Metrics

Vehicle license plate detection provides the primary identifier used for vehicle record linkage in this study. Table 8 shows the hyperparameters for training the CNN of the YOLO models after experimental testing and considering the best performance obtained.

**Figure 30:**
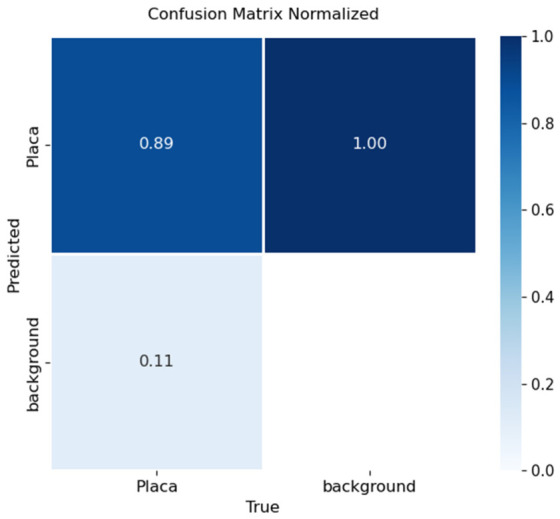
Confusion matrix for license plate detection using YOLOv8x on the validation set.

**Figure 31:**
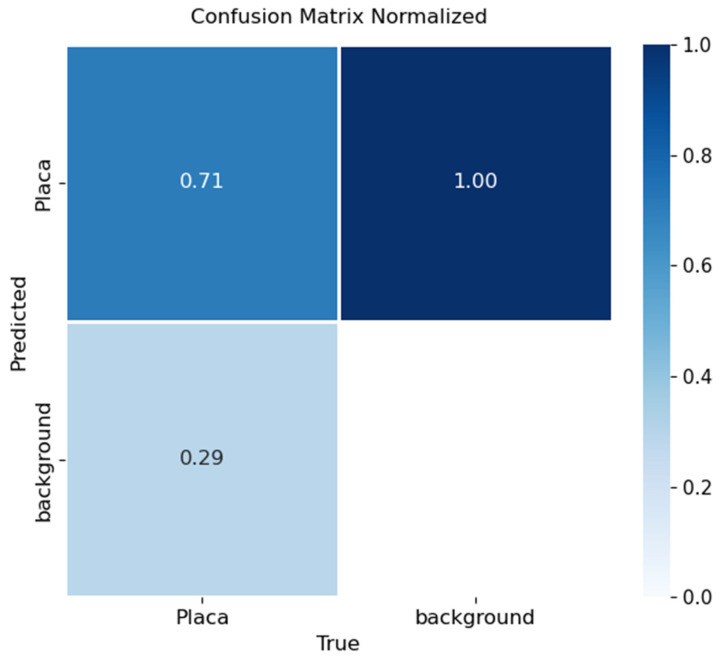
Confusion matrix for license plate detection using YOLOv9e on the validation set.

**Figure 32:**
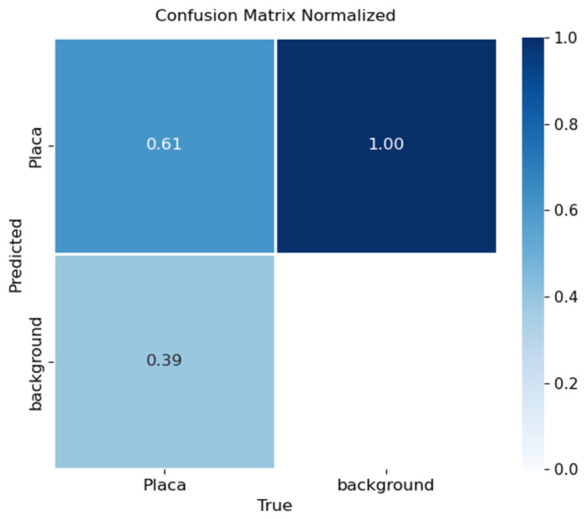
Confusion matrix for license plate detection using YOLOv10x on the validation set.

Performance was assessed through confusion matrices and training curves. Figure 30, Figure 31 and Figure 32 present confusion matrices for YOLOv8x, YOLOv9e, and YOLOv10x.

YOLOv8x (Figure 30): Correctly detected 89% of license plates. Errors (11%) were mainly due to adverse lighting, low resolution, or complex angles. Occasional confusion with visually similar objects (e.g., signs, labels) was observed.

YOLOv9e (Figure 31): Achieved 71% accuracy, showing moderate performance. Around 29% of plates were missed or classified as background. This model exhibited difficulty distinguishing plates under low-contrast or complex backgrounds.

YOLOv10x (Figure 32): Obtained 61% accuracy, with approximately 39% of plates undetected or misclassified. Although functional in certain conditions, its performance decreased in challenging scenarios, particularly under low lighting.

**Figure 33:**
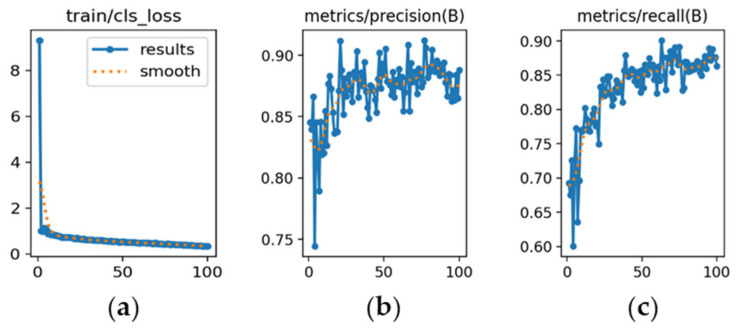
Training curves for license plate detection with YOLOv8x (*x*-axis: epochs): (**a**) classification loss; (**b**) precision; (**c**) recall.

**Figure 34:**
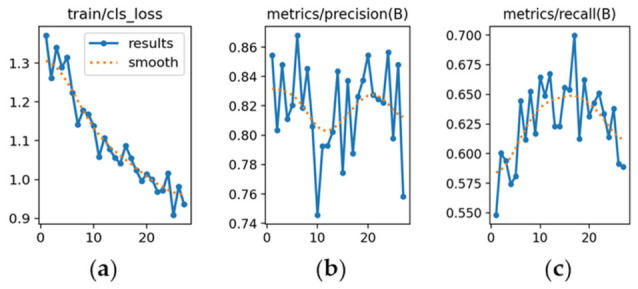
Training curves for license plate detection with YOLOv9e (*x*-axis: epochs): (**a**) classification loss; (**b**) precision; (**c**) recall.

**Figure 35:**
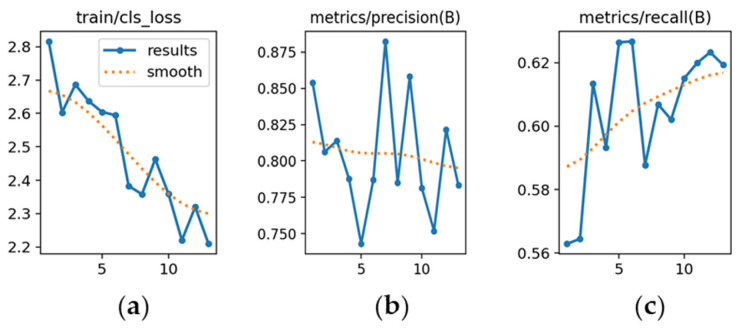
Training curves for license plate detection with YOLOv10x (*x*-axis: epochs): (**a**) classification loss; (**b**) precision; (**c**) recall.

The learning dynamics are shown in Figure 33, Figure 34 and Figure 35.

YOLOv8x (Figure 33): Figure 33a shows a smooth decrease in classification loss, indicating stable convergence. Figure 33b illustrates precision starting at 0.75 (due to transfer learning) and stabilizing between 0.85 and 0.90. Figure 33c shows recall improving from 0.60 to ~0.90, reflecting strong sensitivity.

YOLOv9e (Figure 34): Figure 34a shows fluctuating classification loss with small peaks, starting at ~1.3. Figure 34b indicates precision starting at 0.85 but declining slightly to 0.81, revealing inconsistency. Figure 34c shows recall fluctuating between 0.57 and 0.62, confirming limited sensitivity.

YOLOv10x (Figure 35): Figure 35a reveals a high initial loss (2.8) with irregular convergence. Figure 35b shows precision starting at 0.82 but trending downward, indicating reduced discriminative capability. Figure 35c highlights recall, improving from 0.56 to 0.61, though with significant dispersion.

YOLOv8x provided the most reliable plate detection with AP@0.3 = 0.89. YOLOv9e and YOLOv10x yielded AP@0.3 = 0.71 and 0.61, respectively, with higher variability under low illumination and complex backgrounds. Transfer learning in YOLOv8x contributed to faster convergence and improved stability.

#### 4.4.2. Make/Sub-Model Recognition Predictions

**Figure 36:**
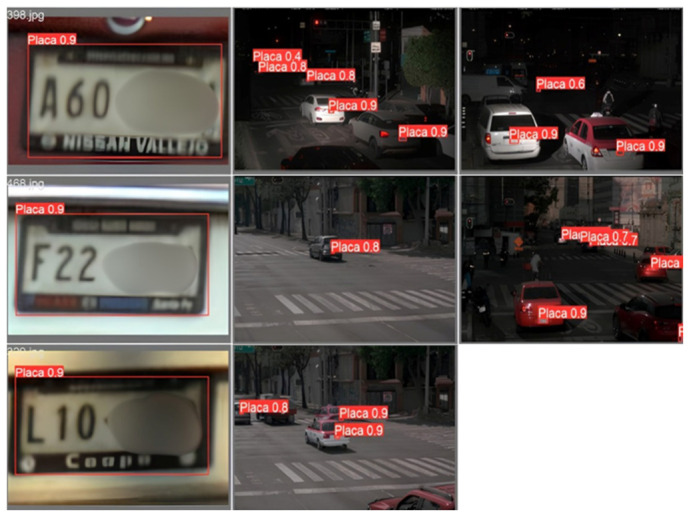
Qualitative examples of license plate localization using YOLOv8x under challenging viewpoints and illumination conditions.

Representative results obtained with YOLOv8x are shown in Figure 36. The detector consistently localized license plates in diverse conditions, including angled views, moving vehicles, and plates under variable illumination. Predictions demonstrated robustness against partial occlusions and reflective surfaces, confirming that YOLOv8x can generalize effectively in real-world applications.

### 4.5. Character Recognition

The final stage of the pipeline focused on recognizing alphanumeric characters from the detected license plates. For this task, PaddleOCR was employed due to its modular architecture and strong performance in multilingual optical character recognition tasks. To improve accuracy, a preprocessing pipeline was implemented to enhance image clarity prior to OCR.

#### 4.5.1. Image Preprocessing

The preprocessing stage aimed to maximize character visibility and reduce background noise:

- Grayscale conversion: Simplified image complexity by removing color information and preserving intensity values.
- Binarization: Applied adaptive thresholding to convert images into binary format, enhancing the contrast between characters and plate background.
- Noise reduction: Median and Gaussian filters were applied to minimize reflections and background interference.
- Contour filtering: Isolated character regions for improved segmentation and recognition accuracy.

This pipeline proved essential in handling challenging conditions such as poor illumination, reflections, dirt, or partial occlusions on license plates.

#### 4.5.2. OCR Recognition

We used PaddleOCR with default detection/recognition settings as described in Section 3.5; here we report the observed OCR performance and typical failure modes.

The processed images were passed to PaddleOCR, which extracted the alphanumeric sequence using its default text detection and recognition modules. We then applied the rule-based post-processing described in Section 3.5 to reduce common confusions (e.g., ‘0’ ↔ ‘O’, ‘1’ ↔ ‘I’/‘l’) and validated the output against plausible alphanumeric formats. When formatting cues were available, the plate was categorized as local, foreign, or taxi.

#### 4.5.3. Results

**Figure 37:**
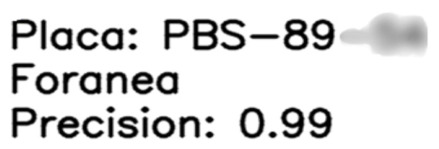
Example OCR outputs after preprocessing with privacy protection (plate region masked; recognized strings partially anonymized).

As shown in Figure 37, PaddleOCR produced stable plate strings across diverse illuminations and viewpoints on the labeled validation samples. Typical failure modes were associated with severe blur, glare, and low-contrast characters. These results indicate that OCR performance is strongly dependent on plate ROI quality and preprocessing, and the extracted strings are suitable for downstream database integration.

These results confirm that the OCR component of the pipeline is both robust and efficient, providing reliable textual data for subsequent database integration and real-world monitoring applications.

For privacy, plate regions are masked and recognized strings are partially anonymized in the examples.

### 4.6. Results Integration

The outputs from all stages—vehicle detection, color recognition, make and sub-model classification, license plate detection, and character recognition—were consolidated into a unified database. This integration ensures that each vehicle is represented by a structured record, enabling efficient data management and retrieval for downstream applications.

Processing and storage. Each processed frame generated a record containing:

- A vehicle record ID (per frame/instance).
- The recognized license plate string.
- The plate type (local, foreign, or taxi).
- The detected vehicle color.
- The identified make and sub-model.
- A timestamp indicating the processing date and time.

The records were stored in a MySQL relational database, allowing robust data consistency and fast queries.

Automation and queries. Data insertion was fully automated to minimize human intervention and errors. Real-time availability was ensured, enabling applications such as traffic monitoring and mobility analytics. The system supports fast queries, for example:

- Retrieving all vehicles detected within a specific time window.
- Filtering by license plate type or recognition confidence.
- Identifying duplicate records for consistency checks.

Routine maintenance tasks include duplicate removal, indexing, and backups to ensure long-term data integrity.

## 5. Discussion

We evaluated the multi-stage pipeline with task-appropriate metrics: for vehicle detection we report precision, recall, and mAP as defined in Section 3.4, while license plate detection is reported at IoU = 0.3 (AP/mAP@0.3) due to the small-object nature of plates and downstream OCR usability. Under this protocol, YOLOv8 offered the most consistent module-level balance (plate-detection AP@0.3 = 0.89; color macro-F1 = 0.88; make and sub-model accuracy ≈ 0.92), while maintaining stable throughput. YOLOv9 slightly improved fine-grained model discrimination but was less stable on plates; YOLOv10 was more tolerant to viewpoint changes yet converged more slowly.

YOLOv8 achieved the most stable convergence across training metrics (classification loss, precision, recall). It offered high accuracy in vehicle detection and robust recognition of specific colors and makes. However, its performance was somewhat sensitive to illumination changes, leading to misclassifications in certain tonal categories such as black, silver, and maroon.

YOLOv9 outperformed YOLOv8 by differentiating visually similar sub-models, such as Nissan Versa and Nissan Tiida, showing its capacity for fine-grained recognition. Nevertheless, precision and recall curves exhibited fluctuations, suggesting that the model was more sensitive to class imbalance and complexity in the dataset.

YOLOv10 achieved slightly lower precision overall but demonstrated resilience under challenging conditions, such as oblique viewpoints and variable lighting. Its classification loss decreased more irregularly compared to the other models, indicating slower convergence and the need for longer or more refined training strategies, particularly for small-object tasks like license plate detection.

In terms of license plate recognition, performance differences were notable: YOLOv8 reached 89% accuracy, while YOLOv9 and YOLOv10 achieved 71% and 61%, respectively. These results confirm that YOLOv8 is better suited for small-object detection tasks when combined with OCR, providing a stronger balance between precision and recall.

Color recognition results highlighted the role of illumination consistency. All models showed strong classification for uniform categories (yellow, taxi, black), while confusion arose in colors with high tonal similarity (red vs. maroon, silver vs. white). This reinforces the importance of including diverse lighting conditions in training datasets to improve robustness.

Overall, YOLOv8 emerged as the most balanced model, offering competitive precision, stable recall, and computational efficiency. YOLOv9 and YOLOv10 demonstrated specific advantages—fine-grained recognition and adaptability under complex viewpoints—but at the expense of stability and small-object accuracy.

The integration of transfer learning, early stopping, and dataset augmentation was critical for mitigating overfitting and accelerating convergence across all experiments. These techniques proved particularly valuable for license plate detection, where plate resolution and environmental conditions represent major challenges.

## 6. Conclusions

This work presented a five-stage modular pipeline combining YOLO-based detectors/classifiers with OCR for multi-attribute vehicle analysis. YOLOv8 provided the most balanced module-level performance across the pipeline, including strong license plate detection (AP@0.3 = 0.89), robust color classification (macro-F1 = 0.88), and make/sub-model recognition (accuracy ≈ 0.92). The complete pipeline, including OCR, achieved approximately 18–22 FPS on recorded traffic videos depending on scene complexity.

Across the models tested, YOLOv8 proved to be the most balanced option, delivering over 90% accuracy in vehicle detection and make recognition, and maintaining the highest performance in license plate recognition (89%) compared with YOLOv9 (71%) and YOLOv10 (61%). YOLOv9 showed strength in distinguishing closely related sub-models, while YOLOv10 demonstrated resilience in challenging scenarios such as oblique viewpoints and poor illumination, albeit with less stable convergence.

The experiments also showed the importance of transfer learning, early stopping, and dataset augmentation, which helped reduce overfitting and improved performance, especially in small-object detection. Training times reflected hardware constraints: YOLOv10 required significantly longer runs on the GTX 2070 GPU compared to YOLOv8 and YOLOv9, which achieved faster and more stable convergence.

Regarding inference performance, YOLOv8m achieved the lowest latency (≈22 ms/frame), followed by YOLOv9m (≈29 ms/frame) and YOLOv10m (≈32 ms/frame). The complete pipeline, including OCR processing, maintained a total per-frame inference time of approximately 45–55 ms, confirming its feasibility for real-time applications such as traffic monitoring, traffic composition analytics, incident analysis support, and mobility management.

The evidence indicates that YOLOv8 is the most suitable candidate for practical deployment, balancing robustness, accuracy, and computational efficiency. Future research may focus on expanding datasets with synthetic data, exploring ensemble strategies, and integrating transformer-based architectures to further strengthen performance under uncontrolled environmental conditions.

## 7. Future Works

Several directions remain open to extend this research. One of the most immediate steps is to increase the size and diversity of the training datasets. Incorporating additional images captured under adverse conditions—such as rain, fog, nighttime illumination, or high vehicle density—would improve the robustness of the models and reduce the impact of class imbalance. Synthetic data generation could also be explored as a complementary strategy to enrich underrepresented classes.

Another promising line is the use of ensemble models, where different YOLO versions or complementary architectures are combined to take advantage of their respective strengths. For instance, YOLOv8 may provide stable detection of license plates, while YOLOv9 or YOLOv10 could contribute to fine-grained classification of make and sub-models.

In parallel, the integration of transformer-based vision models represents an opportunity to capture global context and long-range dependencies, which could mitigate some of the difficulties observed in color recognition and in distinguishing visually similar vehicle categories.

Finally, real-time deployment should be further investigated. Optimizing inference for embedded hardware or edge devices would make the system suitable for applications such as traffic monitoring, mobility analytics, and urban safety applications. Beyond technical performance, future work should also consider aspects such as scalability, privacy, and the interoperability of the recognition system with existing intelligent transportation infrastructures.

## Figures and Tables

**Table 1:** Comparative overview of recent YOLO + OCR and multi-attribute vehicle identification systems.

Ref.	Year	Attributes	Detector	OCR	Dataset	Metrics	Runtime	Limitation
Ammar et al. [9]	2023	Plate + vehicle ID	Multi-stage (YOLO-based)	OCR	Real-world video	Recognition accuracy + runtime	Yes	Focus mainly on LPR
Al-Hasan et al. [10]	2024	Plate	YOLOv8	OCR	Qatar plates	Detection + recognition	Yes	Plate-focused
Gayen et al. [11]	2024	Make/Model	Survey	N/A	N/A	N/A	N/A	Not an integrated pipeline
Tumrani et al. [12]	2020	Multi-attribute (Re-ID)	N/A	N/A	Public benchmarks	Re-ID + attribute metrics	N/A	Not ALPR/not OCR-based
You et al. [13]	2025	Plate	YOLOv8	PaddleOCR	Self-collected	Plate detection + OCR accuracy	Yes	No multi-attribute

**Table 2:** YOLO model variants evaluated in this study and their parameter counts (in millions) by version (v8, v9, v10) [3,4,5].

Model	Parameter (Millions)	Year
YOLOv8n	3.0	2023
YOLOv8s	11.2	
YOLOv8m	25.9	
YOLOv8l	43.6	
YOLOv8x	68.2	
YOLOv9t	2.0	2024
YOLOv9s	7.2	
YOLOv9m	20.1	
YOLOv9c	25.5	
YOLOv9e	58.1	
YOLOv10n	2.3	2024
YOLOv10s	7.2	
YOLOv10m	15.4	
YOLOv10l	24.4	
YOLOv10x	29.5	

**Table 3:** Relational database schema used to store the unified vehicle record (plate text, plate type, color, make/sub-model, and timestamp).

Dato	Description
ID (INT)	Vehicle record identifier (per frame/instance)
Plate (VARCHAR)	Recognized license plate number
Plate type (VARCHAR)	Local, foreign or taxi
Color (VARCHAR)	Detected vehicle color
Make and sub-model (VARCHAR)	Recognized make and sub-model
Date and time (TIMESTAMP)	Processing timestamp

**Table 4:** Task-specific dataset composition and train/validation splits used for each stage of the pipeline (vehicle detection, color, make/sub-model, and license plate detection).

Database	Dataset	Characteristics			
		Images	Class	Class	Resolution
Own	Vehicle detection train Transfer learning train	1500 1938	1	Auto	4008 × 3096 4000 × 2992
	Vehicle detection val Transfer learning val	883 485		Auto	4008 × 3096 4000 × 2992
	Color recognition (train/val)	1760 485	11	Yellow; Blue; White; Brown; Burgundy; Orange; Black; Silver; Red; Green; Taxi	917 × 704 1280 × 960
	Make and sub-model recognition (train/val)	7451 1142	6	Nissan_Versa Nissan_Tiida Chevrolet_Aveo Volkswagen_Seat Volkswagen_Vento Volkswagen_Jetta	917 × 704 1280 × 960
	License plate detection train Transfer learning train	5859 960	1	License plate	917 × 704 1280 × 960
	License plate detection val Transfer learning val	1465 240			

**Table 5:** Training hyperparameters used for vehicle detection across YOLOv8m, YOLOv9m, and YOLOv10m (including transfer learning configuration).

Hyper-Parameters									
Model	Epochs	Pretrained	Image Size	lr0	lrf	Batch	Dropout	IoU	Optimizer
YOLOv8m	100	False	640	1.0 × 10^−6^	1.0 × 10^−4^	8	0.5	0.3	Adam
Transfer learning	100	True	640	1.0 × 10^−6^	1.0 × 10^−4^	8	0.5	0.3	Adam
YOLOv9m	100	False	640	1.0 × 10^−6^	1.0 × 10^−4^	8	0.5	0.3	Adam
Transfer learning	100	True	640	1.0 × 10^−6^	1.0 × 10^−4^	8	0.5	0.3	Adam
YOLOv10m	100	False	640	1.0 × 10^−6^	1.0 × 10^−4^	8	0.5	0.3	Adam
Transfer learning	100	True	640	1.0 × 10^−6^	1.0 × 10^−4^	8	0.5	0.3	Adam

**Table 6:** Training hyperparameters used for vehicle color recognition across YOLOv8m, YOLOv9m, and YOLOv10m.

Hyper-Parameters									
Model	Epochs	Pretrained	Image Size	lr0	lrf	Batch	Dropout	IoU	Optimizer
YOLOv8m	100	False	640	1.0 × 10^−6^	1.0 × 10^−4^	8	0.5	0.3	Adam
YOLOv9m									
YOLOv10m									

**Table 7:** Training hyperparameters used for make and sub-model recognition across YOLOv8m, YOLOv9m, and YOLOv10m.

Hyper-Parameters									
Model	Epochs	Pretrained	Image Size	lr0	lrf	Batch	Dropout	IoU	Optimizer
YOLOv8m	100	False	640	1.0 × 10^−6^	1.0 × 10^−4^	8	0.5	0.3	Adam
YOLOv9m									
YOLOv10m									

**Table 8:** Training hyperparameters used for license plate detection across YOLOv8x, YOLOv9e, and YOLOv10x (including transfer learning configuration).

Hyper-Parameters									
Model	Epochs	Pretrained	Image Size	lr0	lrf	Batch	Dropout	IoU	Optimizer
YOLOv8x	100	True	640	1.0 × 10^−6^	1.0 × 10^−4^	8	0.5	0.3	Adam
Transfer learning	100	True	640	1.0 × 10^−6^	1.0 × 10^−4^	8	0.5	0.3	Adam
YOLOv9e	100	True	640	1.0 × 10^−6^	1.0 × 10^−4^	8	0.5	0.3	Adam
Transfer learning	100	True	640	1.0 × 10^−6^	1.0 × 10^−4^	8	0.5	0.3	Adam
YOLOv10x	100	True	640	1.0 × 10^−6^	1.0 × 10^−4^	8	0.5	0.3	Adam
Transfer learning	100	True	640	1.0 × 10^−6^	1.0 × 10^−4^	8	0.5	0.3	Adam

## Data Availability

Privacy and data governance. The data used in this study were obtained from recorded traffic monitoring videos. No personally identifying information was intentionally collected. License plate information is treated as sensitive; therefore, the dataset is not publicly released. Some illustrative figures may contain partially readable license plate numbers due to the real-world nature of the recorded scenes; however, we minimized exposure where feasible. Access to the data is restricted and available only upon reasonable request for research purposes, subject to privacy and data-use constraints.
